# Knowledge and awareness regarding human papillomavirus and its association with oropharyngeal cancer among the general population in Makkah, Saudi Arabia: a cross-sectional study

**DOI:** 10.3389/fonc.2026.1894082

**Published:** 2026-08-04

**Authors:** Khulud A. Alhazmi, Sulaiman Bani AbdelRahman, Hala Altarawneh, Karem Ibrahem, Bandar Hasan Saleh, Abdulaziz Alsaedi, Ahmad M. Alharbi, Radi Alsafi, Waiel S. Halabi, Mohammed Mufrrih, Mohammad Hassan Albar, Salem Agabawi, Ala A. Azhari, Eman A. Abu-Seer, Mashael S. Alfaifi, Mohammed A. Almatrafi, Rakan Ekram, Tassnym H. Sinky, Mohammed Samannodi, Basem A. Jawa

**Affiliations:** 1 Department of Microbiology and Parasitology, Faculty of Medicine, Umm Al-Qura University, Makkah, Saudi Arabia; 2 Department of Microbiology and Pathology, Faculty of Medicine, Mutah University, Mutah, Al-Karak, Jordan; 3 Department of Clinical Microbiology and Immunology, Faculty of Medicine, King Abdulaziz University, Jeddah, Saudi Arabia; 4 Department of Clinical Microbiology Laboratory, King Abdulaziz University Hospital, Jeddah, Saudi Arabia; 5 Department of Clinical Laboratories Sciences, College of Applied Medical Sciences, Taif University, Taif, Saudi Arabia; 6 Laboratory Medicine Department, Faculty of Applied Medical Sciences, Umm Al-Qura University, Makkah, Saudi Arabia; 7 Department of Optometry, Faculty of Applied Medical Sciences, University of Jeddah, Jeddah, Saudi Arabia; 8 Department of Medical Laboratory Sciences, Faculty of Applied Medical Sciences, King Abdulaziz University, Jeddah, Saudi Arabia; 9 Special Infectious Agents Unit BSL-3, King Fahd Medical Research Center, King Abdulaziz University, Jeddah, Saudi Arabia; 10 Department of Obstetrics and Gynecology, Faculty of Medicine, King Abdulaziz University, Jeddah, Saudi Arabia; 11 Department of Internal Medicine, Faculty of Medicine, King Abdulaziz University, Jeddah, Saudi Arabia; 12 Vaccines and Immunotherapy Unit, King Fahd Medical Research Center, King Abdulaziz University, Jeddah, Saudi Arabia; 13 Department of Epidemiology and Medical Statistics, College of public Health and Health informatics, Umm Al-Qura University, Makkah, Saudi Arabia; 14 Department of Pediatrics, College of Medicine, Umm Al-Qura University, Makkah, Saudi Arabia; 15 Department of Health Administration and Hospitals, Faculty of Public Health and Health Informatics, Umm Al-Qura University, Makkah, Saudi Arabia; 16 Department of Health Promotion and Health Education, College of Public Health and Health Informatics, Umm Al-Qura University, Makkah, Saudi Arabia; 17 Department of Medicine, College of Medicine, Umm Al-Qura University, Makkah, Saudi Arabia; 18 Laboratory and Blood Bank, Alnoor Specialist Hospital, Makkah Health Cluster, Makkah, Saudi Arabia

**Keywords:** awareness, general population, human papillomavirus, knowledge, oropharyngeal cancer

## Abstract

**Background:**

Human papillomavirus (HPV) represents the most prevalent sexually transmitted infection globally and is recognized as a principal etiological agent of anogenital, cervical and oropharyngeal cancers (OPC). Notably, HPV serves as a significant risk factor for OPC, the incidence of which has been rising worldwide in recent years. This study was conducted to assess the level of awareness and knowledge regarding HPV and its association with OPC among residents of Makkah, Saudi Arabia.

**Materials and methods:**

All adults (aged 18 years and over, n = 420) were surveyed cross-sectionally in Makkah, Saudi Arabia. Data were collected using structured and self-administered questionnaires online. The instrument included sociodemographic data, and questions about awareness and knowledge of HPV infection and its association with OPC. Descriptive statistics were used for all the variables and there were multiple choice questions to which the answers were ‘Yes’, ‘No’ and ‘Do not know’.

**Results:**

The adults participated were a total of 420. About half of the respondents (51.7%) were previously heard of HPV and only 21.4% had encountered an awareness campaign about HPV and OPC. Over half (53.6%) correctly identified HPV as not a bacterial infection, while fewer recognized HPV as being contagious (43.1%) and that it could be transmitted through sexual contact (44.3%). Knowledge of HPV prevention was variable; 67.9% recognized that HPV vaccination can reduce the risk of HPV infection, but only 42.4% were aware that vaccines are available to help prevent HPV infection. Also, awareness of the symptoms of OPC and risk factors were inconsistent, with the most common being oral lesions and neck lump, and the most identified risk factors were smoking and alcohol consumption. Most participants (88.1%) reported insufficient knowledge about HPV and OPC and expressed a need for additional information, with social media identified as the preferred strategy for increasing public awareness (38.1%).

**Conclusion:**

The findings demonstrate important gaps in public knowledge and awareness of HPV, its association with OPC, and HPV prevention among adults in Makkah. Targeted educational interventions and public awareness campaigns are needed to improve understanding of HPV transmission, vaccination, and OPC prevention. Given that most participants expressed a need for additional information, digital platforms, particularly social media, may provide an effective approach for delivering public health education.

## 1 Introduction

HPV is a member of the Papillomaviridae family, are small, nonenveloped viruses with double-stranded DNA genomes that encode a limited number of proteins. HPVs are implicated in approximately 5% of all human cancers, including those of the vagina, cervix, vulva, anus, penis, and oropharynx. Based on their oncogenic potential, HPV types are categorized as low-risk (LR-HPV) and high-risk (HR-HPV) genotypes. Among these, HR-HPV types 16 and 18 are the most prevalent globally and account for most HPV-associated malignancies. The oncogenicity of HPV is primarily attributed to the activities of its E6 and E7 proteins, which disrupt critical cell cycle regulators such as retinoblastoma protein (pRB) and p53, facilitating neoplastic transformation and carcinogenesis. Additionally, the E5 protein may also contribute to the tumorigenic process (1).

HPV comprises over than 200 related virus strains, with certain types transmitted through anal, vaginal, or oral sexual contact. There are two types of sexually transmitted HPV: Low-risk (LR) or high-risk (HR)according to their oncogenic risk. HR-HPV types, notably HPV 16 and 18, together with types 31, 33, 35, 39, 45, 51, 52, 56, 58, and 59, are implicated in Numerous types of cancer, with HPV 16 and 18 accounting most of these. Whereas, LR-HPV types are rarely to cause cancer, they may cause benign lesions like throat, oral, anal, or genital warts. In a few cases, respiratory papillomatosis, which is the development of warts in respiratory tract or larynx that may lead to airway obstruction (2). HPV infection is extremely common, with almost everyone who is sexually active contracting the virus within months to a few years of sexual debut, about half of these infections are with HR- HPV types. The Most HPV infections are transient and resolve spontaneously in a year or two due to efficient clearance by the immune system, without developing to malignancy. However, the persistent infection with HR-HPV types can cause cellular changes which could progress from precancerous lesions to invasive cancer, if not treated. HPV is etiologically linked to six distinct cancer types: anal, cervical, oropharyngeal, penile, vaginal, and vulvar cancers. Notably, prophylactic HPV vaccination offers significant protection by preventing infection with oncogenic HPV types, thereby substantially reducing the incidence of HPV-associated cancers and genital warts (2).

Numerous cancers have been linked to infection with specific HPV types such as those of the anogenital, and head and neck regions, especially OPC (3). Several individuals are exposed to oral HPV in their life. Oral HPV infection is more common in older age with about 10% men and 3.6% women infected. Most individuals clear HPV within one to two years, however, HPV infection can persist in some individuals. HPV can infect the mouth and the throat. It usually takes years after being infected with HPV for cancers to develop in the oropharynx (the back of the throat, involving the base of the tongue and tonsils). This is known as OPC (4). OPC, predominantly presenting as squamous cell carcinomas originating from the epithelial lining of the oropharynx, represents a significant global health challenge, with an estimated 99,000 new cases and over 48,000 deaths each year. Recent evidence highlights the importance of HR-HPV infection, specifically HPV types 16 and 18, in pathogenesis of oropharyngeal squamous cell carcinomas (OPSCC). In recognition of these etiological distinctions, the World Health Organization’s 2022 classification now differentiates between the two distinct types of OPSCC: HPV-associated OPSCC (HPV-OPSCC) and HPV-independent OPSCC, with each having a different epidemiological, clinical, histopathological, treatment and clinical outcome profile. HPV-independent OPSCCs are typically associated with older persons of lower socioeconomic status, chronic alcohol and tobacco use, and no link to increased sexual activity, while HPV-OPSCCs are most often observed in younger, socioeconomically advantaged white males with higher levels of sexual activity and limited exposure to alcohol and tobacco (5).Meng et al. (2025) reported that, it is estimated 1,505,394 new HPV-related cancer cases were registered and 755,303 deaths worldwide in 2022, corresponding to an age-standardized incidence rate (ASIR) of 20.9 per 100,000 and an age-standardized mortality rate (ASMR) of 10.2 per 100,000. The study revealed significant regional variation, with Africa having the highest figures in both ASM and ASIR. Cervical cancer remained the principal contributor (ASIR 14.1 per 100,000), despite the head and neck cancers accounted for the largest number of cases (685,204). Notably, there was a significantly greater burden of HPV-related cancers in low- human Development Index (LHDI) countries, with incidence rates 1.3 times greater (and mortality rates almost triple) than in very high- human Development Index (VHDI) countries. The authors note the incidence of cervical cancer is decreasing in most countries, contrasted by the incidence of head and neck cancer is increasing among women and anal cancer is increasing among both men and women, underscoring the need to develop targeted interventions based on the cancer burden in the different regions (6). Cervical cancer is the fifth most common cancer worldwide in females, with around 604,000 new cases and 280,000 deaths expected in 2024. Cervical cancer incidence and mortality rate is higher in the low- and middle-income countries, compared to high-income countries. In 26 countries (predominantly in sub-Saharan Africa, some of South and Central America, Melanesia and some countries in South-East Asia), cervical cancer remains the main cause of cancer related death among females. These differences highlight inequities, primarily due to poor access to national HPV vaccination programs, cervical cancer screening and treatment, and other social and economic factors that influence health (7). HPV also is estimated to account for over 90% of cervical cancers and anal, around 70% of vulvar and vaginal cancers, and 60% of penile cancers. While OPC have traditionally been associated with alcohol and tobacco use, recent studies suggest that 60%-70% of OPC may be attributable to HPV infection. Many of these cases are likely due to the synergistic effects of drinking alcohol, smoking and HPV (8). The real incidence of HPV infection is likely substantially underestimated in public health statistics as most cases are asymptomatic and therefore remain unrecorded in public health data sources. In the example of HPV in Girona, Spain, from 2010 to 2014, only approximately 33% of the estimated number of actual infections was registered by the public health system. This major underreporting has important consequences, since many public health strategies and decisions concerning the allocation of resources are based on reported figures which may result in suboptimum prevention efforts (9).

The incidence of OPC is rising among both women and men worldwide, with the highest rates observed in high-resource regions such as Northern Europe and North America, where the incidence can reach up to 8 per 100,000 males. Globally, OPC incidence is markedly higher in men compared to women (4.0 versus 0.79 per 100,000), and recent trends indicate a disproportionate burden among older white male populations (10). The observed rise in OPC incidence is largely attributed to an increase in HPV-associated OPC, particularly among middle-aged and older adults. In the United States, there has been a notable escalation in HPV-related OPC cases between 2002–2006 and 2014–2018, with incidence rates increasing from 17.1 to 22.0 per 100,000 among vaccine-ineligible men aged 45–64 years, and from 17.5 to 28.4 per 100,000 among men over 65 years of age (10, 11).

Recent epidemiological evidence indicates that HPV remains an important public health concern in Saudi Arabia. A recent systematic review and meta-analysis reported the prevalence of HPV in Saudi Arabia to be comparable to the global estimates, there was some variation from region to region with HPV-16 being the most common genotype, followed by HPV-18 and other high-risk genotypes (12). Moreover, a recent national laboratory-based study showed a trend of rising high-risk HPV infection rates from 2016 to 2024, with HPV-16 being responsible for around 25% of HPV-positive cases, and significant regional differences in prevalence throughout the Kingdom. The results continue to show the presence of the oncogenic HPV genotypes in Saudi Arabia and the need to increase public awareness, vaccination and prevention measures (13).

In addition to preventing anogenital and cervical cancers, the HPV vaccine has been developed to also prevent OPC and may help to lower risk of these cancers. CDC recommends HPV vaccination for persons aged 11–12 years and suggests catch up vaccination for those who have not been vaccinated until they reach age 26. Decisions about vaccination for adults (27 to 45) should be made on a case-by-case basis as there is a high risk of previous exposure to HPV. Ideal protection is obtained when the vaccine is given prior to any exposure to HPV. Vaccination, consistent and correct condom use, and dental dams can reduce risk for HPV transmission, as alcohol consumption and tobacco use are also shown to increase the risk for OPC, especially when combined, are also important behavioral strategies (14).

Although population-based incidence data for HPV-associated oropharyngeal cancer in Makkah are currently limit, the city’s large resident population and annual influx of millions of visitors make public awareness of HPV-associated diseases particularly important. Improving public awareness may contribute to the promotion of preventive measures and early recognition of HPV-related diseases. Therefore, this study aimed to assess the knowledge and awareness of HPV and its association with oropharyngeal cancer among adults residing in Makkah. The findings will provide important baseline data that may guide future research, educational initiatives, and public health strategies aimed at improving awareness and prevention of HPV-associated diseases.

## 2 Materials and methods

### 2.1 Study description

This cross-sectional study was conducted among the general adult population of Makkah, Saudi Arabia. The study was designed to assess their knowledge and awareness toward the association between HPV and OPC. Data were collected using an online, self-administered questionnaire designed to evaluate participants’ demographics, HPV knowledge and awareness, sources of information about HPV associated OPC.

### 2.2 Sample size and participants recruitments

The sample size was calculated using the Raosoft calculator (https://raosoftcalculator.com/), according to the population size of Makkah 2,763,316 (worldpopulationreview.com). The sample size required for the study was 385 with a 95% confidence interval and 5% margin of error. The final sample size was increased by 10% to allow for non-response and incomplete data. After data collection, 4 incomplete questionnaires were excluded, resulting in a final sample size of 420 participants.

The following steps were taken to reduce potential biases associated with the distribution of the online survey:

#### 2.2.1 Broad recruitment

Widespread dissemination of the survey link on various platforms and social media, and use of QR code for survey link in the face-to-face survey to reach a diverse audience.

#### 2.2.2 Anonymous participation

Respondents were assured of anonymity in order to get honest and unbiased answers.

#### 2.2.3 Clear instructions

Detailed instructions were given to ensure that the participants fully understood the questions and process of the survey.

#### 2.2.4 Randomized distribution

An attempt was made to distribute the survey at different times and days to get response from different demographic groups.

#### 2.2.5 Inclusion criteria

Specific inclusion and exclusion criteria were used to ensure the relevance and appropriateness of respondents.

#### 2.2.6 Monitoring response

Google form setting was changed to allow each person to submit one response only.

### 2.3 Eligibility criteria

The study was conducted in Makkah, Saudi Arabia, and targeted adults aged 18 and above who are residing in the city. Participants were males and females, residents of Makkah at the time of the study, of Saudi and non-Saudi nationality. However, children under 18 years and the residents of Makkah outside the city were not allowed to participate.

### 2.4 Data collection

Data were collected using a self-administered online questionnaire. To ensure content validity, the questionnaire was reviewed by two virology experts. The pilot study with 30 subjects was also conducted to test the questions and the time required to answer the questions; however, this phase was not included in the final analysis. The participants’ knowledge regarding HPV-associated OPC and their awareness were assessed using multiple-choice questions with answers of “Yes”, “No” or “I do not know”. The dataset contains:

- Demographic variables
- Source of information
- HPV knowledge questions
- HPV transmission questions
- Treatment/vaccination questions
- OPC risk factors
- Cancer risk factors
- OPC symptoms
- Awareness questions
- Preferred educational methods

Each participant accessed the survey through a direct link shared via an instant messaging platform (SMS, Email, WhatsApp, Instagram, Face book, Twitter and other social media platforms) using Google Forms. After completing the questionnaire, they were instructed to submit the web form, which then transferred the responses to a spreadsheet (Excel, Microsoft Corp.) for analysis.

### 2.5 Ethical consideration and informed consent

The study was conducted in accordance with the principle of the declaration of Helsinki.

Also, the Biomedical Research Ethics Committee at Umm Al-Qura University, Makkah, Saudi Arabia, approved the study (Approval No. HAPO-02-K-012-2024-12-2407). Informed online consent was obtained from all participants before they proceeded with the questionnaire. Furthermore, an online statement outlining the study’s purpose was provided prior to data collection. To maintain the confidentiality of participants’ information, all surveys were conducted anonymously.

### 2.6 Statistical analysis

Data collected from the completed questionnaires were entered into Microsoft Excel (Microsoft Corporation, Redmond, WA, USA) for data management and analysis. Descriptive statistical analyses were performed, and categorical variables are presented as frequencies and percentages.

## 3 Results

### 3.1 Demographic characteristics

The demographic information of the 420 participants is shown in **Table 1**. Participants ranged in age from 18 years to over 60 years, with most participants (74.5%) aged 18–25 years. Most participants were female (373; 88.8%), while the remainder were male. The majority were Saudi nationals, 391 (93.1%), with 29 (6.9%) being non-Saudi. In terms of marital status, 321 participants (76.4%) were single, while 99 (23.6%) were married. Regarding educational level and employment status, 263 participants (62.6%) held a bachelor’s degree, and 292 (69.5%) were students (**Table 1**).

**Table 1:** Demographic characteristics of the participants, including gender, age, nationality, marital status, educational level, and employment status.

Item	Category	% (number)
Gender	Male	11.2 (47)
	Female	88.8 (373)
Age	18-25	74.5 (313)
	26-45	12.4 (52)
	46-60	10.2 (43)
	>60	2.9 (12)
Nationality	Saudi	93.1 (391)
	Non-Saudi	6.9 (29)
Marital status	Single	76.4 (321)
	Married	23.6 (99)
Educational level	Uneducated	2.4 (10)
	Less than high school	3.8 (16)
	High school	27.6 (116)
	Bachelor	62.6 (263)
	Higher education	3.6 (15)
Employment field	Student	69.5 (292)
	Non-employee	8.1 (34)
	Medical field	9.3 (39)
	Non-medical field	13.1 (55)

### 3.2 Sources of information about HPV and OPC

**Figure 1:**
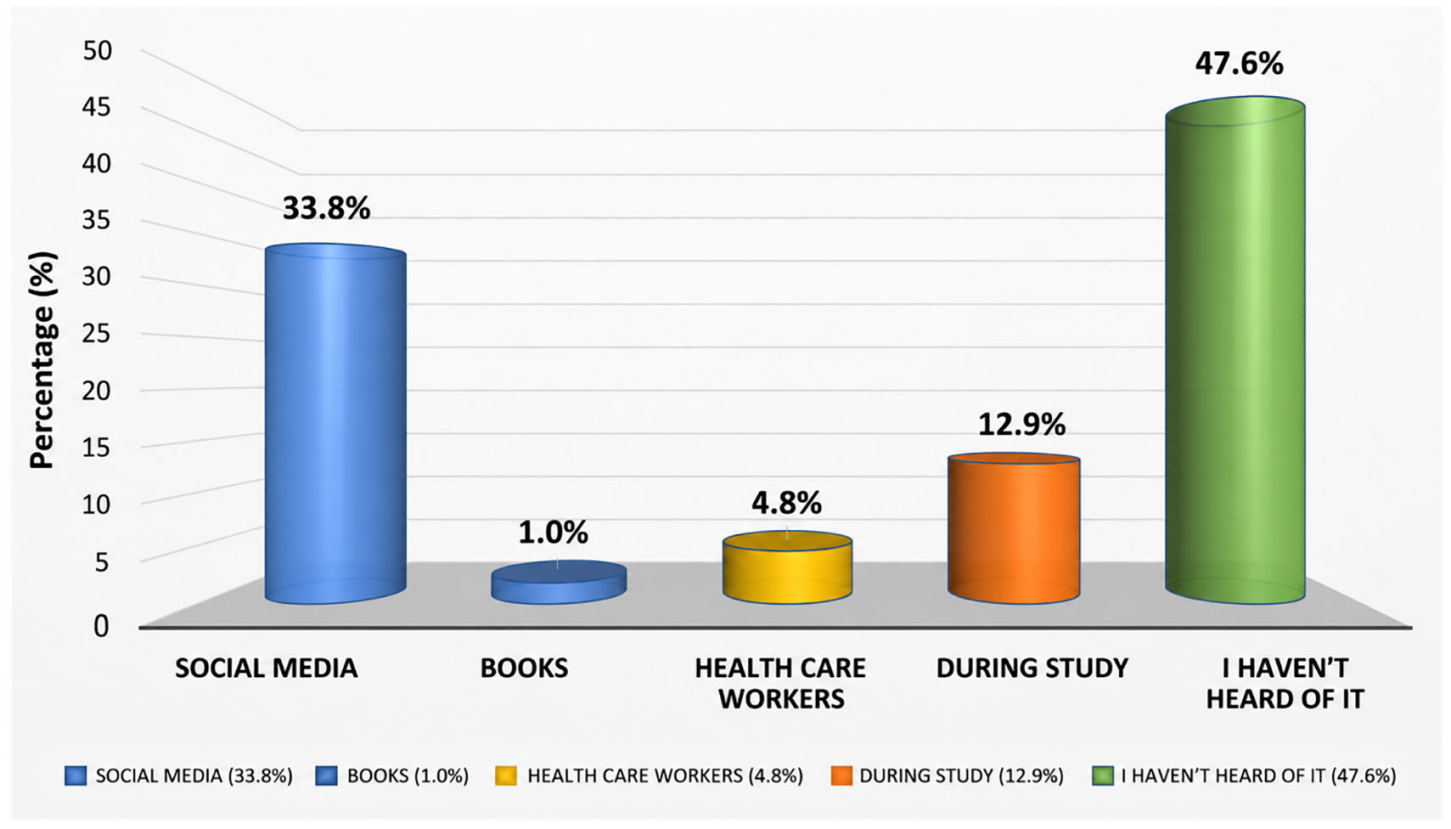
Participants’ sources of information about the association between HPV and OPC.

**Figure 1** presents the sources from which participants obtained information about the association between HPV and OPC. Most participants (33.8%) reported obtaining their information from social media, followed by information acquired during their studies (12.9%), healthcare workers (4.8%), and books (1.0%). However, 47.6% of the participants had not heard of the association between HPV and OPC.

### 3.3 General knowledge about HPV infection

When asked whether HPV is a bacterial infection, 53.6% of the participants answered it correctly, as HPV is not a bacterial infection, and 63.3% answered that it is a viral infection. Only 24.8% correctly identified HPV as a common infection rather than a rare one. Furthermore, 43.3% reported that some people might not have signs or symptoms of an HPV infection. In addition, 48.8% knew that certain HPV strains can cause both genital warts and cancer. Only 32.4% of participants believed that HPV infection and early cellular changes may occur without noticeable symptoms. Additionally, 36.4% understood that the types of HPV responsible for genital warts are different from those that cause cancer. Just 31.9% were knew that having genital warts caused by HPV does not necessarily mean the individual will develop cancer. Only 17.6% correctly recognized that HPV does not cause HIV/AIDS, whereas 56.0% were unsure and 26.4% incorrectly believed that it does (**Table 2**).

**Table 2:** Participants’ general knowledge about HPV infection.

Item	Category	% (number)
HPV is a bacterial infection	Yes	10 (42)
	No	53.6 (225)
	I do not know	36.4 (153)
HPV is a viral infection	Yes	63.3 (266)
	No	5 (21)
	I do not know	31.7 (133)
HPV is a very rare infection	Yes	21.2 (89)
	No	24.8 (104)
	I do not know	54 (227)
Some infected people have no symptoms. Therefore, people may be exposed to infection without knowing it	Yes	43.3 (182)
	No	7.4 (31)
	I do not know	49.3 (207)
HPV may cause warts and cancer	Yes	48.8 (205)
	No	6.2 (26)
	I do not know	45 (189)
Symptoms of HPV-related cancer may not appear until advanced stage	Yes	32.4 (136)
	No	10.2 (43)
	I do not know	57.4 (241)
HPV types causing warts differ from those causing cancer	Yes	36.4 (153)
	No	11 (46)
	I do not know	52.6 (221)
Having genital warts does not necessarily mean developing cancer	Yes	31.9 (134)
	No	12.6 (53)
	I do not know	55.5 (233)
HPV can cause HIV/AIDS	Yes	26.4 (111)
	No	17.6 (74)
	I do not know	56 (235)

### 3.4 General knowledge about HPV transmission

Approximately 43.1% of participants knew that HPV is a contagious infection, and 49.3% understood that it can be transmitted from one infected person to another. In addition, 33.6% of them acknowledged that genital HPV is one of the most prevalent sexually transmitted infections. Approximately 44.3% knew that HPV could be transmitted through sexual contact, and 22.6% were knew that it could be transmitted through skin-to-skin contact, especially during sexual activity. Only 10.0% correctly answered that HPV is not usually transmitted through bodily fluids or secretions. Although HPV can be detected in bodily fluids such as semen, it is primarily transmitted through contact with infected skin or mucous membranes (**Table 3**).

**Table 3:** Participants’ general knowledge about HPV transmission.

Item	Category	% (number)
Is HPV contagious?	Yes	43.1 (181)
	No	11.7 (49)
	I do not know	45.2 (190)
Can HPV be transmitted from one infected person to another?	Yes	49.3 (207)
	No	8.8 (37)
	I do not know	41.9 (176)
Genital HPV is one of the most common sexually transmitted infections	Yes	33.6 (141)
	No	13.1 (55)
	I do not know	53.3 (224)
Can HPV be transmitted through sexual contact?	Yes	44.3 (186)
	No	8.3 (35)
	I do not know	47.4 (199)
HPV may be spread by skin contact with someone who has the infection.	Yes	22.6 (95)
	No	24.5 (103)
	I do not know	52.9 (222)
Can HPV spread through bodily fluids or secretions of an infected person?	Yes	41 (172)
	No	10 (42)
	I do not know	49 (206)

### 3.5 HPV treatment and vaccination

**Figure 2:**
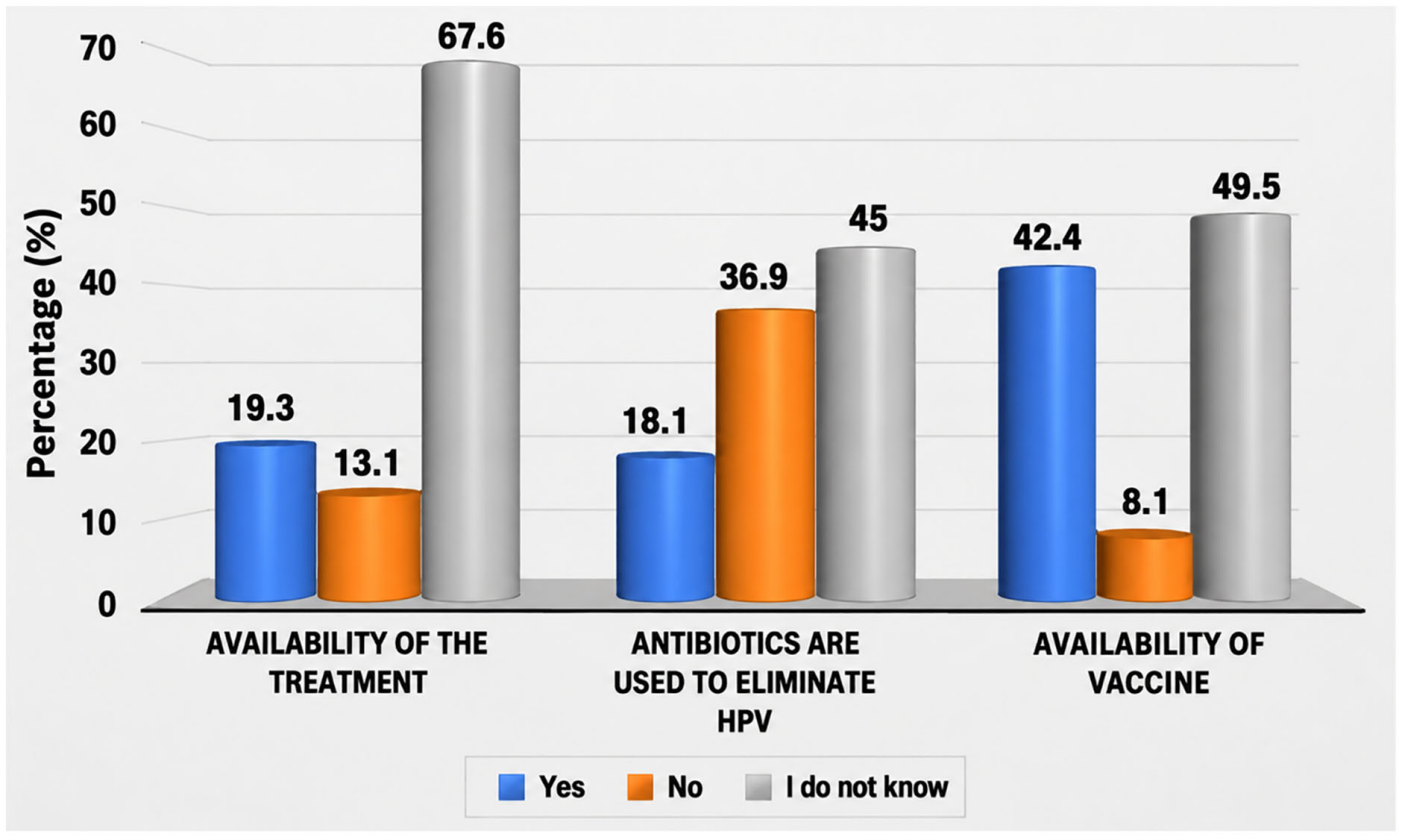
Participants’ general knowledge of HPV treatment and vaccination.

Only 19.3% of participants correctly recognized that there is no antiviral treatment that eradicates HPV infection and that available treatments target HPV-related diseases rather than the virus itself. In addition, 36.9% knew about the ineffectiveness of antibiotics for HPV. Only 42.4% knew that vaccines are available to help protect against HPV infection (**Figure 2**).

### 3.6 Knowledge of factors that may increase the risk of developing OPC

**Figure 3:**
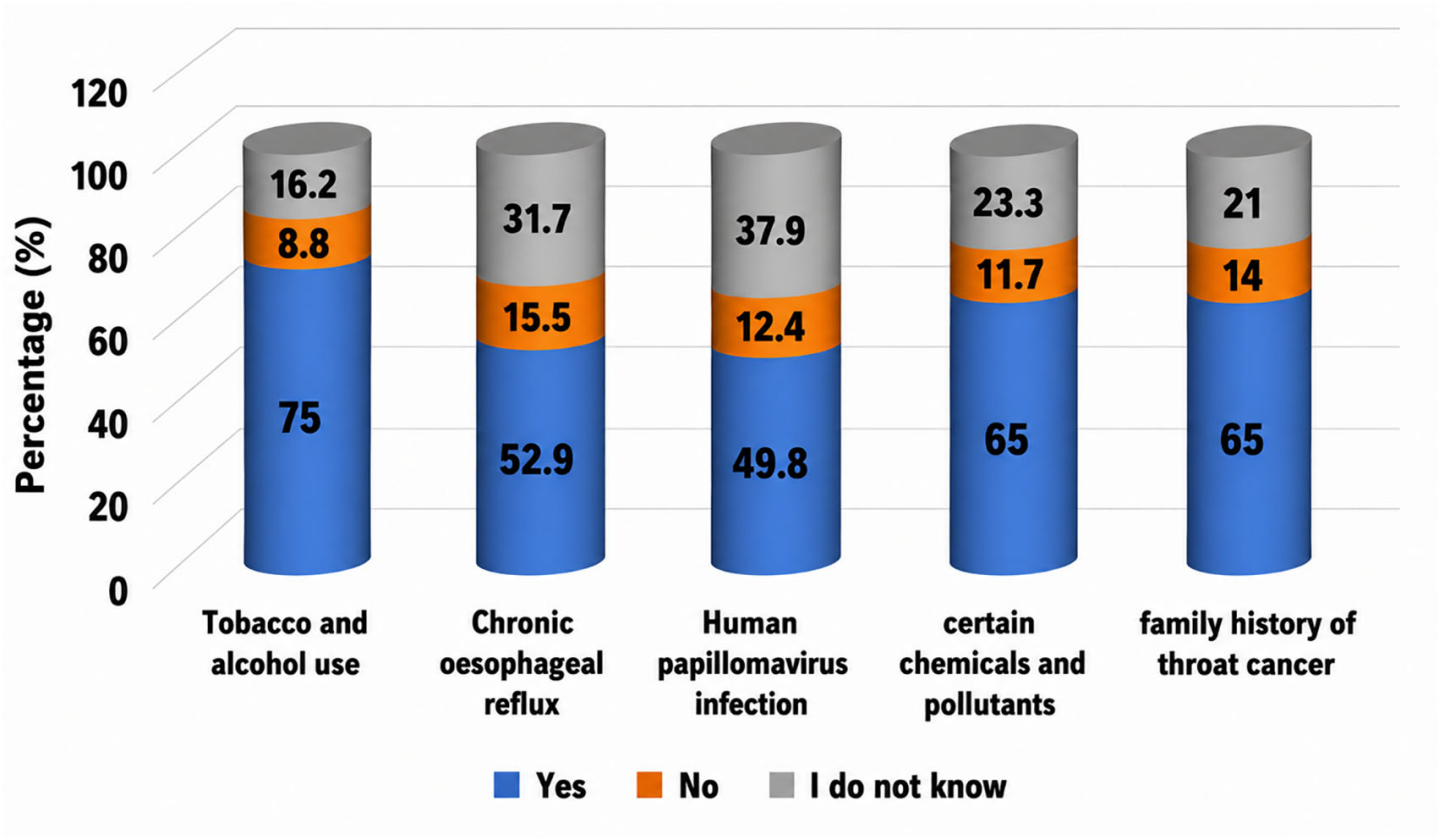
Participants’ knowledge of factors that may increase the risk of developing OPC.

Regarding risk factors for developing OPC, approximately three-quarters of participants identified tobacco and alcohol use as risk factors for OPC. Furthermore, 65% agreed that exposure to certain chemicals and pollutants, as well as a family history of throat cancer, were associated with an increased risk of developing the disease. In addition, 52.9% believed that chronic esophageal reflux was a risk factor, and 49.8% identified HPV infection as a risk factor (**Figure 3**).

### 3.7 Knowledge about common factors that increase the risk of cancer

About 71.9% of participants recognized that excessive alcohol consumption is a common risk factor for cancer. Additionally, participants identified several other contributing factors, including smoking 78.1%, chewing tobacco 73.1%, tonbak use 67.1%, marijuana use 58.1%, poor oral hygiene 46.4%, HPV infection 49%, a family history of cancer 68.6%, and Only 19.3% identified exposure to sunlight as a risk factor for certain cancers, such as skin cancer. Moreover, Sixty-five percent of participants correctly recognized that consuming fruits and vegetables is not associated with an increased risk of cancer (**Table 4**).

**Table 4:** Participants’ knowledge about some common factors that increase the risk of cancer.

Item	Category	% (number)
Excessive alcohol consumption	Yes	71.9 (302)
	No	7.6 (32)
	I do not know	20.5 (86)
Smoking	Yes	78.1 (328)
	No	6.4 (27)
	I do not know	15.5 (65)
Chewing tobacco	Yes	73.1 (307)
	No	7.1 (30)
	I do not know	19.8 (83)
Tonbak use	Yes	67.1 (282)
	No	8.3 (35)
	I do not know	24.5 (103)
Marijuana use	Yes	58.1(244)
	No	8.3 (35)
	I do not know	33.6 (141)
Poor oral hygiene	Yes	46.4 (195)
	No	20.5 (86)
	I do not know	33.1 (139)
HPV infection	Yes	49 (206)
	No	10.7 (45)
	I do not know	40.2 (169)
Family history of cancer	Yes	68.6 (288)
	No	11.9 (50)
	I do not know	19.5 (82)
Exposure to sunlight	Yes	19.3 (81)
	No	48.8 (205)
	I do not know	31.9 (134)
Consumption of fruits and vegetables	Yes	11.2 (47)
	No	65 (273)
	I do not know	23.8 (100)

### 3.8 Knowledge of warning signs and symptoms of OPC

About 56% of participants recognized a persistent sore throat as one of the warning signs of OPC. Additionally, 71.4% identified difficulty swallowing or the sensation of a lump in the throat as a symptom, while 61% were knew that hoarseness or voice changes could also be indicative. Chronic cough or coughing up blood was recognized by 69% of participants, earache or hearing loss by 42.9%, unexplained weight loss by 57.9%, and pain in the neck or throat by 66%. These symptoms were recognized by participants as potential warning signs of OPC (**Table 5**).

**Table 5:** Participants’ knowledge of some warning signs and symptoms of OPC.

Item	Category	% (number)
Persistent sore throat	Yes	56 (235)
	No	13.1 (55)
	I do not know	31 (130)
Difficulty swallowing or a feeling of a lump in the throat	Yes	71.4 (300)
	No	8.3 (35)
	I do not know	20.2 (85)
Hoarseness or changes in voice	Yes	61 (256)
	No	10.5 (44)
	I do not know	28.6 (120)
Chronic cough or cough with blood	Yes	69 (290)
	No	8.1 (34)
	I do not know	22.9 (96)
Earache or hearing loss	Yes	42.9 (180)
	No	14 (59)
	I do not know	43.1 (181)
Unexplained weight loss	Yes	57.9 (243)
	No	10 (42)
	I do not know	32.1 (135)
Pain in the neck or throat	Yes	66 (277)
	No	7.1 (30)
	I do not know	26.9 (113)

### 3.9 Awareness and perceptions of HPV infection

Regarding participants’ awareness and perception of HPV infection, about half (51.7%) had previously heard of HPV, while 48.3%had not. A majority (67.9%) understood that HPV vaccination can help reduce the risk of HPV infection. However, only 21.4% had encountered an awareness campaign or received information about the link between HPV and OPC, while 78.6% had not. Additionally, most participants (88.1%) perceived their knowledge and awareness of HPV and OPC to be insufficient and expressed a need for more information (**Table 6**).

**Table 6:** Participants’ awareness and perceptions of HPV infection.

Item	Category	% (number)
Before today, have you heard of HPV?	Yes	51.7 (217)
	No	48.3 (203)
Can HPV vaccination reduce the risk of HPV infection?	Yes	67.9 (285)
	No	5.7 (24)
	I do not know	26.4 (111)
Have you seen an awareness campaign about HPV and OPC?	Yes	21.4 (90)
	No	78.6 (330)
Do you think your information is insufficient about HPV and you need awareness and extra information?	Yes	88.1 (370)
	No	11.9 (50)

### 3.10 Strategies to increase awareness

**Figure 4:**
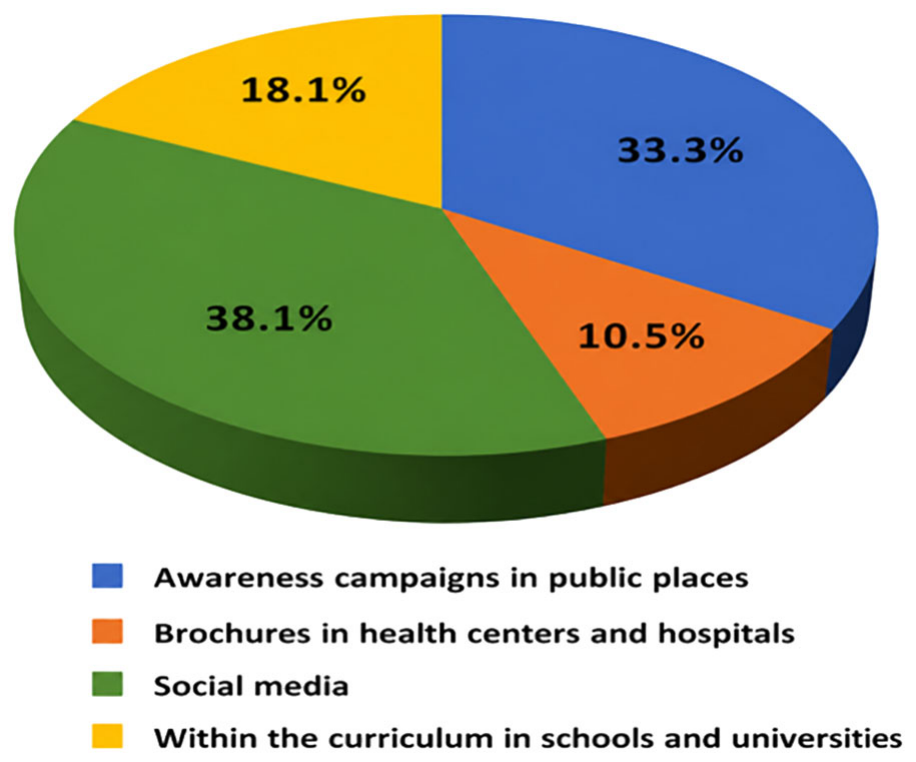
Participants’ opinions on the best ways to increase public awareness and knowledge regarding the association between HPV and the risk of OPC.

Participants shared their opinions on the most effective ways to increase public awareness and knowledge about the association between HPV and the risk of OPC. Social media was the most recommended method, cited by 38.1% of participants. This was followed by public awareness campaigns in public places (33.3%), incorporating information into school and university curricula (18.1%), and distributing brochures in health centers and hospitals (10.5%) (**Figure 4**).

## 4 Discussion

The findings revealed important gaps in participants’ knowledge and awareness of HPV, its association with OPC, and HPV transmission.

In the present study, participants’ general knowledge and awareness about HPV infections were inadequate. Only 53.6% of participants correctly identified that HPV is not a bacterial infection. Correspondingly, a recent investigation conducted among college students in North Carolina reported that 36.7% of respondents accurately recognized that human papillomavirus is not a bacterial pathogen (15). Furthermore, 48.8% of participants knew that certain HPV strains can cause genital warts and several forms of cancer.

Furthermore, participants’ general knowledge about the spread of HPV was insufficient. Approximately 43.1% of participants in the present study knew that HPV is a contagious infection. Similarly, a study conducted among female secondary school students in Nigeria reported that 43.1% of participants demonstrated limited overall knowledge about HPV, including an understanding of its contagious nature (16). Approximately 44.3% of participants recognized sexual contact as a mode of HPV transmission. Similarly, a recent study conducted among women in the Medina region reported that only 35.9% of participants recognized that HPV is sexually transmitted (17). Moreover, only 22.6% of participants aware that HPV could also be transmitted through skin-to-skin contact with an infected individual, mainly during sexual activity. These findings underscore a gap in awareness and knowledge of HPV transmission.

The results indicate limited knowledge regarding available treatment options for HPV-related diseases and the availability of vaccines that help in the prevention of HPV infection. Only 19.3% of participants knew that treatments are available for health conditions caused by HPV. In addition, 36.9% of participants in this study were aware that antibiotics are ineffective against HPV infection, as they target bacterial pathogens rather than viral agents such as HPV. In this study, only 42.4% of participants knew that vaccines are available to help protect against HPV infection. These findings further highlight the general population’s limited awareness of HPV vaccination. Likewise, a recent survey of non-clinical staff of community-based HIV/AIDS services organizations in the southern United States found that fewer than half of the respondents (39–48%) had a positive opinion about the ability of the HPV vaccine to prevent oral, anal, penile, vulvar and vaginal cancers (18).

Several types of human papillomavirus (HPV) vaccines have been developed, and early vaccination is recommended to ensure protection prior to potential exposure to the virus. Gardasil-9 (9vHPV), currently distributed in the United States, confers immunity against nine HPV types (58, 52, 45, 33, 31, 18, 16, 11 and 6). Notably, the U.S. Food and Drug Administration (FDA) has approved Gardasil 9 for an expanded indication that includes the prevention of oropharyngeal and other head and neck cancers attributable to HPV types 58, 52, 45, 33, 31, 18 and 16. Previously, the quadrivalent (Gardasil, 4vHPV) and bivalent (Cervarix, 2vHPV) vaccines were also licensed by the FDA. Importantly, all HPV vaccines protect against HPV types 16 and 18, which are responsible for most HPV-related cancers (19, 20). Thus, the HPV vaccine is strongly recommended for the prevention of HPV-associated cancers. However, many participants were uncertain about the vaccine’s role in reducing the risk of HPV-related OPC.

OPC risk is known to be associated with alcohol and tobacco consumption, and the combined effects of alcohol and tobacco use are greater than the effects of either alone (21). The study was conducted to assess the level of awareness of the risk factors associated with OPC among the respondents. Approximately three-quarters of participants identified alcohol and tobacco use as risk factors for OPC. In contrast, only 49.8% recognized HPV infection as a potential risk factor for this type of cancer. This difference points to a significant lack of awareness within the public about the contribution of HPV to OPC, as there is growing evidence that HPV contributes substantially to the global burden of OPC.

The International Agency for Research on Cancer (IARC) has determined that cigar and pipe smoking is strongly linked to a higher risk for, and causation of, cancers of the lungs and upper aerodigestive tract, such as the oral cavity, hypopharynx, oropharynx, larynx and esophagus. Furthermore, there are known causal associations between the use of smokeless tobacco and cancer of the esophagus, oral cavity, and pancreas (22). Additionally, decades have been spent studying tobacco products, and they are known to be significant carcinogenic agents. This study assessed participants’ awareness of factors associated with an increased risk of cancer. 78.1% of the respondents identified cigarette smoking as a risk factor, and 73.1% identified chewing tobacco as a factor that increases the risk of cancer development. In addition, 67.1% knew that tonbak is another risk factor for cancer, and 58.1% knew that using marijuana is a potential risk factor. These results indicate that participants’ awareness of traditional tobacco-related risk factors appeared relatively high. However, there is a need to improve awareness of less-recognized carcinogenic factors, particularly HPV infection, tonbak use, and marijuana use, for more comprehensive cancer prevention.

The findings revealed a satisfactory level of knowledge regarding several signs and symptoms of OPC. Specifically, 71.4% recognized difficulty swallowing or a sensation of a lump in the throat as a potential symptom. Furthermore, 69% recognized persistent coughing or coughing up blood as a symptom, while 66% identified pain in the neck or throat as a possible warning sign. In addition, 61% recognized hoarseness or voice changes as possible symptoms.

Recognition of other warning signs was lower, with 56.0% identifying persistent sore throat, 57.9% identifying unexplained weight loss, and 42.9% recognizing earache or hearing loss as possible symptoms of OPC. A study performed in Karachi in 2014 also revealed that the awareness level of the non-medical professionals about the signs and symptoms of throat cancer was found to be very low; less than 50% of the respondents had good awareness in this regard (23).

Sixty-seven percent (67.9%) of respondents indicated that they were aware of HPV vaccination, which reduces the risk of infection with the most common HPV types. This relatively acceptable level of awareness is contrasted with the results of a recent study conducted by Alghalyini et al. (2024), which reported that only 36.2% of the participants knew of the existence of the HPV vaccine (24). The findings of both studies, however, continue to underscore a need for continued community education on HPV vaccination, as knowledge and perception play a crucial role in raising HPV vaccine coverage and reducing the risk of HPV related diseases. About half of the respondents (51.7%) also heard of HPV. Likewise, an HPV knowledge study among Thai females in 2011 revealed that just 40% knew about HPV (25). The results indicate that while awareness could be different in different communities and at different levels, there is still inadequate awareness regarding HPV in many communities. Additionally, 47.6% of the participants had no idea that HPV is a risk factor for OPC. Verhees et al., 2021 conducted a study in which only 29.2% of the participants were aware of the association between HPV and OPC (26). The results illustrate the ongoing lack of public understanding of the link between HPV and OPC.

Consistent with this, recent studies among Saudi dental students have shown knowledge and awareness gap. Alsharif and Alsahafi (2024) found that Saudi dental students in the Western Region had acceptable awareness of HPV and its association with oropharyngeal squamous cell carcinoma and HPV vaccination while significant knowledge gap was observed (27). Whereas Alsalhani et al. (2024) reported dental students and interns presented a good but less than optimal awareness and knowledge regarding HPV and its prevention (28). These findings suggest the knowledge and awareness gaps concerning HPV-and its associated OPC not only among the general population but also among some healthcare-related student populations in Saudi Arabia, highlighting the need for targeted educational interventions.

In this study, only 21.4% reported having seen an awareness campaign or information specifically relating to the relation between HPV and OPC. Similarly, Fathi et al. (2025) underscored the critical need of more public health campaigns for awareness creation about HPV and its association with various cancer types (29). The findings highlight the importance of implementing specific educational programs to address the gaps in the community’s current knowledge, and to promote preventive behaviors. Furthermore, most of the participants (88.1%) believed their knowledge and awareness of HPV was inadequate and they would like to learn more. This is in agreement with the study conducted by Alanazi et al. (2024) which reported a low level of knowledge among the participants about HPV (30). The study in Ghana in 2022 indicated that 68.2% of them had low knowledge about HPV (31). Overall, the results further highlight the lack of HPV knowledge in various regions and populations. These results suggest continued educational initiatives and targeted awareness campaigns focused on HPV prevention and health risks to bridge these knowledge gaps and facilitate informed decisions around HPV prevention.

Social media, as the best way to raise awareness and knowledge of the HPV-OPC relationship, was recommended by 38.1% of the participants. This was followed by the idea of public awareness campaign in community areas and public places, with 33.3% of the participants supporting this idea. These preferences reflect the perceived value of the use of existing platforms and community outreach to disseminate information and raise awareness of HPV related health risks. The result supports that of Lama et al. (2021), who found that there was a positive correlation between using social media and HPV awareness (32). The benefits of digital platforms in disseminating health information and improving public knowledge are further supported by other evidence.

## 5 Strengths and limitations

There are some limitations in this study that need to consider for interpretation of the findings. The first was the convenience sampling method in which the sample was selected mostly from the online social media platforms. This is also likely to have resulted in an under-representation of individuals with limited internet access, low digital literacy or low levels of education, potentially reducing the representativeness and generalizability of the results to the population of Makkah as a whole. Second, there is the possibility of recall, reporting, and social desirability bias with self-administered questionnaires, since the patient may have misunderstood what they were asked or may have failed to report the information accurately. Thirdly, volunteer bias may have occurred as people with greater interest in health-related topics or those who are more active online may have been more likely to volunteer. Furthermore, there were more female participants in the study than male participants. This sex imbalance might create a selection bias and should be taken into consideration while interpreting findings as it could restrict the generalizability of findings to the overall adult population of Makkah. Lastly, the questionnaire measured knowledge and awareness of HPV and OPC but did not measure other factors related to HPV knowledge and awareness. Hence, results must be considered taking these limitations into account.

Despite these limitations, this study has numerous strengths. To our knowledge, although previous studies have evaluated knowledge of HPV infection and HPV vaccination among the general population or women in Makkah, no published study has specifically assessed knowledge and awareness of HPV and its association with OPC among the general adult population in Makkah, Saudi Arabia. This study was conducted to address this gap in the literature. Also, the sample size was relatively large (420) and a structured questionnaire was used to assess knowledge, awareness, perceived educational needs, and awareness strategies desired. Results are informative baseline data that can be used in developing future public health education programs and awareness campaigns to enhance HPV knowledge and preventions.

## 6 Conclusion

This study revealed that resident of Makkah knowledge and awareness about HPV and its linkage with OPC were inadequate. There was limited knowledge on the participants about HPV transmission, treatment and HPV vaccination. While some OPC symptoms were well known, there were gaps in knowledge for other symptoms. Social media as the preferred platform for raising public awareness and knowledge of the HPV/OPC connection was identified.

The results highlight the need to raise awareness and knowledge about HPV and its link with OPC in the community to promote prevention at the community level. Healthcare institutions should play a pivotal role in establishing comprehensive educational strategies, such as the use of digital platforms, including SMS, mobile apps, websites, videos, and social media, for specific demographic groups. Other efforts include setting up information booths at community events, schools, and health fairs and partnering with cancer networks, community groups, and hospitals to maximize outreach. Encouraging open dialogue between healthcare providers and the public may further improve awareness of the relationship between HPV and OPC.

## Data Availability

The original contributions presented in the study are included in the article/supplementary material. Further inquiries can be directed to the corresponding author.
